# Serial Correlations of Partial Body Weight and Feed Intake in Crossbred Cattle

**DOI:** 10.3390/ani16030402

**Published:** 2026-01-28

**Authors:** Georgette Pyoos, Michiel Scholtz, Michael MacNeil, Mokgadi Seshoka, Frederick Neser

**Affiliations:** 1 Agricultural Research Council, Animal Production Institute, Private Bag X 02, Irene 0062, South Africa; 2 Department of Animal Science, University of the Free state, P.O. Box 339, Bloemfontein 9300, South Africa; 3 Delta G, 145 Ice Cave Road, Miles City, MT 59301, USA; 4 Northern Cape Department of Agricultural, Land Reform and Rural Development, Private Bag X9, Jan Kempdorp 8550, South Africa

**Keywords:** crossbred, beef, production, environment

## Abstract

Feeding behavior in cattle affects feed efficiency, which is important for increasing the profitability of production while simultaneously reducing the environmental impact. Over a six-year period, indigenous beef cows (Afrikaner, Bonsmara, Nguni) were crossed with indigenous and exotic (Angus, Simmental) sires in a hot and arid area, to produce 15 breed groups. After weaning, the bull calves were fed in a feedlot setting wherein daily feed intake and partial body weight were measured. The serial correlations of daily feed intake and partial body weight on consecutive days were estimated for each animal. Analyses of variance for the z-transformed serial correlations of daily feed intake and partial body weight were conducted. The linear model included the fixed effect of test group comprising pen and date at the beginning of the test and a fixed breed group effect. The average serial correlation of daily feed intake (r = 0.10) was interpreted to suggest that a test period of 36 days was sufficient to achieve 80% average accuracy for the animals being tested. The average serial correlation of partial body weight was very high (r = 0.94). Thus, there seems little need to average values over days to achieve an accurate estimate of the weight of an animal at any specific point in time. Variation among animals in the serial correlation of daily feed intake indicates differences in feeding behavior over time, but this variability was not related to breed composition. The results indicate that a test period of 36 days is sufficient to achieve 80% accuracy of the mean for daily feed intake of the animals being tested.

## Simple Summary

An applied question of importance in performance testing of beef cattle is the number of days for which data needs to be recorded in order to achieve a pre-specified accuracy of the mean, particularly for feed intake. However, many estimates of the necessary test period are based result from part–whole relationships between a short test period and a longer one. The objective of this study was to estimate the number of days required to produce a reasonably accurate record of feed intake individually fed beef bulls that does not rely on this part–whole relationship. The average serial correlation of daily feed intake (r = 0.10) was interpreted to suggest that a test period of 36 days was needed to achieve 80% average accuracy for the mean feed intake of an animal being evaluated. Because the serial correlation of partial body weight was very high (r = 0.94), there is little need to average values over days to achieve an accurate estimate of an animal’s partial body weight at any specific point in time.

## 1. Introduction

Feed consumption is essential for survival because it replenishes an animal’s energy and nutrient stores [1]. Feeding behavior in cattle affects feed efficiency, which is important for increasing the profitability of production while simultaneously reducing the environmental impact [2]. In beef production, understanding and managing feed is essential for optimal production [3]. Cattle that can eat comfortably and without stress are more likely to consume the amount of feed they need to attain their genetic potential for growth [4]. It is currently believed that a 42-day test length is sufficient to accurately characterize the feed intake of a beef animal [5]. Recently developed automated technologies can be used to measure daily feed intake (DFI) of individual animals efficiently. These include the upgraded Calan^®^ Gate System (American Calan, Northwood, NH, USA), the newer GrowSafe^®^ Systems, Ltd. (Airdrie, AB, Canada) and the Insentec^®^ Systems (Marknesse, The Netherlands). All these systems use electronic scales and radio frequency identification ear tags to record individual feed intake records in real time [6].

There is substantial day-to-day variation in feed consumption by cattle, and, thus, the number of consecutive days over which it is recorded to produce an accurate measurement for evaluation of individual animals has been a topic of previous research with taurine cattle (e.g., [7,8,9]). Many of these studies make use of the correlation between the average of measurements recorded for a shorter test period with measurements recorded for a longer test period. Using this approach suffers from problems with autocorrelation [10]. Serial correlation occurs when a variable has a natural sequence, such as daily feed intake measured over multiple consecutive days. A serial correlation of zero implies independence of feed intake from one day to the next. A positive serial correlation with a 1-day lag indicates an animal that consumes above-average amounts of feed on one day will again do so on the next. Conversely, a negative serial correlation indicates cyclical feeding behavior.

In the context of beef cattle breeding, the probable producing ability (MPPA) is a well-known tool for the evaluation of individual cows (e.g., [11,12]). Early calculation of MPPA was based on the correlation of adjacent records in addition to estimates of interclass correlation derived from variance components [13]. A cow’s MPPA for weaning weight is calculated from the weaning weights of calves she has previously produced, the number of her progeny with records and the correlation among the repeated records [14]. The accuracy of the most probable producing ability for a cow depends on the correlation among the annually repeated weaning weights of her calves. In a similar manner, a potential use of the serial correlations of feed intake and partial body weight is to estimate the number of days these traits must be recorded to achieve a pre-specified accuracy for the average DFI of animals being evaluated.

The other component of feed efficiency is animal performance, customarily calculated by periodic recording of body weight and computing its rate of increase. Historically, bi-weekly weighing cattle over a 70-day test period has been recommended to provide a measure of their average daily gain for use in calculation of feed efficiency [15]. Problematically, weighing cattle with a conventional chute and scale system imposes a degree of stress [16,17,18] and is labor-intensive. However, a weighing scale cojoined with a watering device can facilitate recording body weight in real time [19]. However, this technology did not achieve widespread use until systems to capture partial body weight (PBW) in real time were developed [20,21]. Measures of PBW may not be entirely sufficient predictors of the weight of an entire animal under some conditions but are certainly sufficient for the calculation of feed efficiency [22].

Therefore, the objective of this study was to use the serial correlations among records of average daily feed consumption and PBW to assess the number of consecutive days over which recording is necessary to achieve a predetermined accuracy of the mean over a prescribed test period. Optimizing the length of time over which feed intake and partial body weight are recorded can result in the evaluation of more animals at less cost relative to excessively long test periods. Further analyses were attempted to partition the estimates of the serial correlation obtained for the individual animals to assess potential breed-specific differences in feeding behavior and growth.

## 2. Materials and Methods

This study made use of data from a crossbreeding trial that was conducted at the Vaalharts research station near Jan Kempdorp in the Northern Cape Province of South Africa. The Vaalharts research station is centrally located in South Africa at 27°57′19″ South and 24°50′41″ East at an altitude of 1175 m. It is in an area with sandy red soil underlaid by lime rock [23]. The veld type is mixed Tarchonanthus veld, Veld type No 16b, 4 [24]. The research station has a recommended carrying capacity of 10 ha/Large Stock Unit. The climate at the Vaalharts research station is characterized by hot summers and cold winters with frost being a common occurrence. The highest monthly average temperature is around ±32 °C and is experienced during December and January. The average precipitation is 440 mm per annum of which 88% is from thunderstorms during the summer months from October to April.

Afrikaner, Bonsmara and Nguni cows (Sanga types) were mated with Afrikaner, Bonsmara, Nguni, Angus and Simmentaler (exotic) bulls in all possible combinations [25]. This created 15 different genotypes. The calves were weaned over a period of six years from 2015 to 2020. Cows that were used in this study either were from the Vaalharts Research Station or were purchased from farms in the Free State, Northern Cape, North West, Eastern Cape, Limpopo, and Mpumalanga provinces, as well as from Namibia. Cattle would typically graze native vegetation throughout the year. However, the cattle that originated from the Free State may have grazed on maize stowage over the winter before their arrival at Vaalharts. The data was collected according to the approved standard operating procedures of the National Beef Recording and Improvement Scheme in South Africa, which is accredited with the International Committee for Animal Recording (ICAR).

The cows of each breed were stratified by age, weight, and estimated breeding values and assigned to mating groups within strata to avoid the possibility of uneven genetic merit of cows mated to any breed of bull. With the exception that some of the Afrikaner cows that were pregnant when they were purchased, each bull was used across the three breeds of dam, and there was connectedness of sires across years. At least two bulls of each breed were assigned to a specific mating group. In year 1, single sire mating was used, whereas in years 2 and 3 multiple sire mating was used, and paternal parentage is therefore unknown. The unknown paternity precludes testing the significance of genetic differences among breeds using the within-breed additive genetic variance. The data resulted from mating Afrikaner, Bonsmara, Nguni, Angus and Simmentaler bulls to Afrikaner, Bonsmara, and Nguni cows over a six-year period.

In total 212, 324, 303, 179 and 234 calves were sired by Afrikaner, Nguni, Bonsmara, Angus and Simmentaler sires, respectively. There were 182, 633 and 437 calves produced by Afrikaner, Nguni and Bonsmara cows, respectively. The breeding season, which ran from December to February, occurred on natural veld, and all calves were raised by their dams from birth through weaning at approximately 205 days of age. Birth dates were recorded, and calves were weighed within 48 h of birth. Each year, all calves were weaned and weighed on the same day. It should be noted that calves are born in spring and weaned in the autumn of the next year.

After weaning, the bull calves were transported from Vaalharts to Irene at weekly intervals in groups of 25–28 according to their weight. Upon arrival in Irene the animals are allocated to pens according to their body weight in accordance with the mandate of the institutional animal ethics committee. The pens were equipped with technology to measure the feed intake of individual animals (GrowSafe, Lenexa, KS 66219, USA). The system of allocation of animals to pens resulted in partial confounding of breed groups and contemporary groups. Thus, it was necessary to assume that breed and contemporary groups that did not interact. This also made the detection of breed group effects, as described later, less likely because the marginal sums of squares for breed groups were conditioned on the contemporary group effects. It should be noted that because data from crossbred animals were included in this study the genetic effects of breed were completely cross-classified with pens. They were given a period of 2 weeks to adapt to the facilities and diet before the commencement of any data collection.

Raw data was edited to standardize the length of time over which the trial occurred each year for all the breed groups. Outliers were identified as residuals having more than 3 standard deviations from zero after preliminary analysis of the data, and the data from these animals was discarded. This editing resulted in the removal of 14 animals from the dataset. Estimates of the serial correlations of DFI and PBW were calculated for each animal. The infrequent pairs of data for which the record from one day was missing were discarded from the data. The time series was not de-trended prior to analysis.

In general, correlations are not normally distributed. Thus, the serial correlations were transformed to *z*-statistics [26].

Fisher’s *z*-transformation is as follows:

$$z=\ln\left(\frac{1+r}{1-r}\right)/2.$$

Wherein *r* designates the estimated product-moment correlation, and ln indicates a natural logarithm. For example, if the Pearson correlation coefficient between two variables was found to be *r* = 0.55, then the corresponding *z*-statistic (*z_r_*) would be: *z_r_* = *ln*((1 + 0.55)/(1 − 0.55))/2 = 0.618. Because the *z*-statistics are normally distributed [27], inferences regarding the correlations can be made using analysis of variance procedures. The z-transformed values of the serial correlations were analyzed using PROC MIXED of the SAS™ System for Windows (Version 8.2, ©1999–2001 by SAS Institute Inc., Cary, NC, USA). The lower bound (*lb*) of a 95% confidence interval for *z* is $z_{lb}=z-\sqrt{\frac{1.96}{N-3}}$, and the upper bound (*ub*) of the 95% confidence interval is $z_{ub}=z+\sqrt{\frac{1.96}{N-3}}$, wherein *N* = the number of observations. The linear model included the fixed effect of test group comprising pen and date at the beginning of the test and a fixed breed group effect. The residual effects from this model were assumed random. The accuracy of an n-day long test for feed intake was calculated from back-transformed values of *z*:

$$r=\frac{e^{\left(2z\right)}-1}{e^{\left(2z\right)}+1}$$

as follows:

$$Accuracy=\;\frac{nr}{(1+\left(n-1\right)r}$$

A subsequent analysis was conducted to estimate the repeatability of DMI, also using PROC MIXED, wherein the fixed effects were augmented with the linear effect of days on test and the interaction of days on test with breed group. In addition, a random effect was fit for animal (a) identification number. This random effect was assumed to be distributed as (0,$I\sigma_{a}^{2}$) which is a simplification of the true distribution of the animal effects due to the unknown relationships among animals that results from unrecorded paternity.

## 3. Results

Large variation was observed in the weaning weights of the different crosses between years. For example, Sanga- and exotic sired calves had the same 205-day corrected weaning weight (171 kg) in the summer of 2015/16, which was very hot (average maximum summer temperature 32.2 °C) with well below average season rainfall of only 302 mm. In contrast, the summer of 2016/17 was cooler (31.1 °C) and wetter (469 mm), and the weaning weights of the Angus and Simmentaler sired calves were 27 kg heavier than the Sanga sired calves.

Analyses of variance for the z-transformed serial correlations of DFI and PBW are shown in Table 1. Denominator degrees of freedom were 332 and 198, respectively. This difference in degrees of freedom between traits here is due to missing data. The data was lost due power outages frequently experienced in South Africa due to load shedding. The test groups differed significantly (*p* < 0.01) in the patterns of feeding behavior and growth as documented by their effects on the z-transformed serial correlation estimates. The lack of significant breed group effects leads us to treat all the animals as generic cattle in further presentation of the results. This also suggests that the length of a centralized test in which feed efficiency is to be evaluated need not vary according to breed group.

Average estimates for the z-transformed serial correlation of DFI and PBW for each animal were 0.1003 and 3.0506, respectively. Using the formula below, these estimates back to correlation coefficients were 0.100 and 0.941, respectively. For comparison to other studies repeatability of DFI was also calculated from the ratio of animal variance to phenotypic variance from the analysis as shown in Table 2. Variance components of the z-transformed serial correlation of DFI for animals and residuals were 0.005680 and 0.07258, respectively. Thus, the measure of the intraclass correlation among the repeated records of the animals was 0.073, slightly less than the back-transformed estimate of the serial correlation.

**Figure 1:**
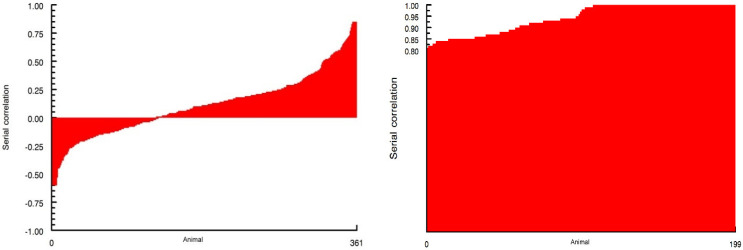
Distribution of serial correlation coefficients for feed intake (**left** panel) and partial body weight (**right** panel) recorded daily.

The 95% confidence interval for DFI was 0.095 < z < 0.106, and for PBW it was 3.041 < z < 3.061. Figure 1 shows the distributions of the correlation coefficients back-transformed from the z-statistics for feed intake and PBW, respectively. The variation in day-to-day DMI is remarkable. Apparently, a sizable number of animals have cyclical patterns of feeding behavior, while others are more consistent over time.

A question of some importance in the performance testing of beef cattle is the number of days for which data needs to be recorded in order to achieve a pre-specified accuracy of the mean. Accurately calculating and predicting phenotypes in growing animals is, in part, informed by the number of days over which the trait is measured [28,29]. Feed intake is a crucial data point required to capture as much of the variability as possible with feed efficiency models. It is vital that intake records are collected with high degrees of accuracy. For example, a 10% increase in feed intake may potentially enhance profits by 43% [30].

**Figure 2:**
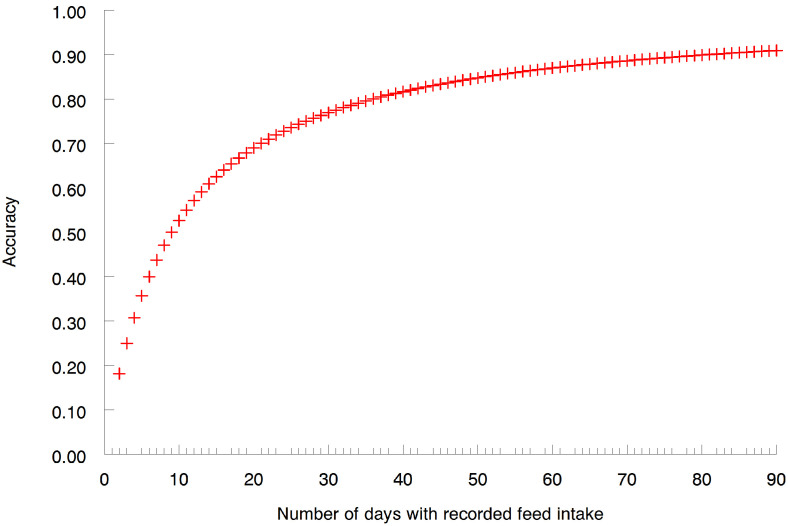
Accuracy (+) of the mean of daily feed intake records as a function of the number of days on which feed intake was recorded.

As shown in Figure 2 is the accuracy of the average of m repeated daily records for DFI based on an estimated serial correlation of 0.10 (i.e., the value from these data). Highlighted in Figure 2 is the number of days (36) over which feed intake records need to be accumulated to achieve an arbitrarily chosen accuracy of 0.8 for an average animal based on the estimated serial correlation. Calculating the number of days needed to achieve this accuracy using serial correlation estimates for the upper and lower limits of the 95% confidence interval indicates a range from 34 to 39 days on test. If the estimate of repeatability (0.073) from the variance components were used in this calculation, then 51 days of feed intake records would be sufficient to achieve an average accuracy of approximately 0.8. These estimates of the required duration for collection of DMI during the postweaning period are near the lower end of the range in similar estimates that have been previously reported [5,7,8,9]. If a greater level of average accuracy were desired, to ensure the accuracy for more animals attained the 80% threshold, a perhaps much longer test would be required. However, shorter test periods facilitate evaluation of a greater number of animals with a fixed capacity in the testing facility and a lower per animal cost for data collection. In contrast, human behavior allows for a period of 2 to 6 days being sufficient to estimate their nutrient intakes with good accuracy (r = 0.8) [31].

## 4. Discussion

It has been suggested that, to have an accurate value for feed intake, either as total feed intake or average DFI, feed intake should be collected on a continuous basis during the testing or evaluation period [32]. The current industry-standard recommendation is that a 42-day test length is sufficient to collect accurate feed intake data [1]. Other research supports test periods that are 35 to 56 days long [5,7,33,34]. These earlier recommendations were based on the relationship of the proportion of variation in average DFI over a longer test interval that was explained by the feed intake records that were recorded over a shorter period of time. This methodology is flawed because (1) it assumes the accuracy of the longer period of data collection is 1.0 and (2) it makes use of a part–whole relationship between the period being evaluated and the entirety of the test. The methodology used in the present study is different in that it does not rely on a part–whole relationship nor assume a perfectly accurate mean value; yet it also supports the industry-standard recommendation. The current study is unique in that it is the only study of its type in which Sanga cattle are represented.

The amount a specific character fluctuates from day to day varies with the trait that is being measured. It is evident in this study that PBW was much more consistent from one day to the next than was feed intake. The remarkably high serial correlation implies that there is little need to average measurements of PBW from successive days of recording to obtain a point estimate of it as, for example, may be desired to obtain a mid-test weight for the calculation of residual feed intake. This result is unsurprising in that the cattle had ad libitum access to a diet calibrated to provide for a constant rate of growth during the test period, and the weight from the previous day makes up a large proportion of the weight on the current day. Additionally, this result does not inform the decision as to the length of an evaluation period that is required to measure any particular trait or index that is derived from body weight records such as average daily gain.

The autocorrelation (equivalent to what is called serial correlation herein) has also been used as an indicator of resilience or equivalently robustness and the opposite of plasticity [35]. The serial correlation of milk yield deviations was lowly heritable and insignificant genetic correlations with both longevity and average test-day milk yield. Further, at least for milk production, this measure of plasticity appears to be independent of phenotypic performance. Based on the present results relative to DFI, it might be suggested that the breed groups represented were all similarly resilient to the environmental conditions that existed during this study.

## 5. Conclusions

The highly variable serial correlation of DFI indicates marked differences among animals in their feeding behavior over time and consequently suggests the optimum feeding period may be animal-specific. However, it does not appear these differences are related to the breed composition of the animals. The average serial correlation of DFI was interpreted to suggest that a test period of 36 days was needed to achieve 80% average accuracy of the mean value for the animals being tested. The similarity and very large estimate of the serial correlation of PBW indicate that the values from individual days are quite useful. Thus, there is little need to average values over days to achieve an accurate estimate of PBW at any specific point in time. It is important to note that this is a very important practical finding, as it provides hard data supporting the period of time needed to characterize the average daily feed consumption of an animal while allowing redundant weighing of animals to be avoided, both of which may be significant for reducing the cost to beef breeders for evaluation of their animals in centralized tests.

## Figures and Tables

**Table 1:** Analyses of variance for z-transformed serial correlations of feed intake and partial body weight of cattle representing 15 breed groups that could be used for beef production in South Africa.

Source	Numerator Degrees of Freedom	Feed Intake		Partial Body Weight	
		F-Value	Probability	F-Value	Probability
Test group *	14	2.37	<0.01	3.53	<0.01
Breed group (B)	14	1.08	0.37	0.98	0.47

* The test groups differed significantly (*p* < 0.01) in the patterns of feeding consumption and growth.

**Table 2:** Analyses of variance for feed intake and partial body weight of cattle representing 15 breed groups that could be used for beef production in South Africa.

Source	Degrees of Freedom		Feed Intake		Partial Body Weight	
	Numerator	Denominator	F-Value	Probability	F-Value	Probability
Test group	14	337	2.26	<0.01	144.62	<0.01
Breed group (B)	14	337	0.64	0.83	1.56	0.09
Days on test (D)	1	16,897	5.68	0.02	8922.36	<0.01
B × D	14	16,897	1.01	0.44	2.01	0.01

## Data Availability

The raw data supporting the conclusions of this article will be made available by the authors upon request.
